# How imagery changes self-motion perception

**DOI:** 10.1016/j.neuroscience.2015.01.021

**Published:** 2015-04-16

**Authors:** Y. Nigmatullina, Q. Arshad, K. Wu, B.M. Seemungal, A.M. Bronstein, D. Soto

**Affiliations:** a Academic Department of Neuro-otology, Division of Brain Sciences, Imperial College, Charing Cross Hospital, Fulham Palace Road, London W6 8RF, UK; b Memory and Attention Laboratory, Division of Brain Sciences, Department of Medicine, Imperial College London, Charing Cross Campus, Fulham Palace Road, London W6 8RF, UK

**Keywords:** EOG, electrooculography, SPV, slow phase velocity, VOR, vestibular–ocular reflex, imagery, vestibular, self motion perception, decision making

## Abstract

Imagery and perception are thought to be tightly linked, however, little is known about the interaction between imagery and the vestibular sense, in particular, self-motion perception. In this study, the observers were seated in the dark on a motorized chair that could rotate either to the right or to the left. Prior to the physical rotation, observers were asked to imagine themselves rotating leftward or rightward. We found that if the direction of imagined rotation was different to the physical rotation of the chair (incongruent trials), the velocity of the chair needed to be higher for observers to experience themselves rotating relative to when the imagined and the physical rotation matched (on congruent trials). Accordingly, the vividness of imagined rotations was reduced on incongruent relative to congruent trials. Notably, we found that similar effects of imagery were found at the earliest stages of vestibular processing, namely, the onset of the vestibular–ocular reflex was modulated by the congruency between physical and imagined rotations. Together, the results demonstrate that mental imagery influences self-motion perception by exerting top-down influences over the earliest vestibular response and subsequent perceptual decision-making.

## Highlights

• Imagined self-motion differentially modulates vestibular processing.
• Differential modulation affects both high- and low-order vestibular processing.
• Congruent and incongruent imagery have opposing effects.
• Modulation reported is specific to mental imagery and not an attentional bias.

## Introduction

Mental imagery allows us to re-experience information recalled from memory across multiple sensory modalities (Kosslyn et al., 2001; Anema et al., 2012). Mental imagery and perception interact, such that imagining visual information can influence detection of visual targets in the environment (Farah, 1985; Craver-Lemley and Arterberry, 2001; Pearson et al., 2008; Anema et al., 2012). This is likely due to overlapping neuronal substrates engaged during perception and imagery across different sensory modalities (Kosslyn et al., 2001), (Kosslyn et al., 2001; Yoo et al., 2003). For instance, imagining listening to music activates similar brain regions as listening to music (Zatorre et al., 1996; Herholz et al., 2012). Beyond perception, mental imagery can also influence action. Common cerebral structures are activated during motor imagination and active execution (Jeannerod, 1994; Jeannerod and Frak, 1999) and motor imagery can facilitate movement and spinal reflexes (Bonnet et al., 1997; Hale et al., 2003; Li et al., 2004; Aoyama and Kaneko, 2011).

The vestibular sense is sometimes referred to as the sixth sense and has a number of critical functions, both low-level such as stabilizing gaze through the vestibular–ocular reflex (VOR) and also higher level, for instance, self-motion perception. To date, understanding of the interaction between mental imagery and self-motion perception is scant. A prior study showed that visual imagery can influence the low-level vestibular reflex when participants experience self-rotation (Barr et al., 1976; Jones et al., 1984). For instance, visually imagining an earth-fixed target during rotation enhances the VOR output whereas imagining a head-fixed target suppresses it (Barr et al., 1976). Critically, these experiments required visual imagery rather than vestibular (self-motion) mental imagery.

To understand how vestibular mental imagery changes self-motion perception, we had participants seated on a motorized chair in the dark and asked them to imagine themselves self-rotating prior to the onset of the physical chair rotation on each trial. The chair rotated at a velocity that increased exponentially and the observers were required to identify the rotation direction. We therefore assessed the influence of imagery contents on both the early vestibular reflex and on later stages associated with self-motion-related perceptual decision making. The response latency (and hence the velocity attained by the chair) was taken as a measure of the perceptual vestibular threshold (Cutfield et al., 2011; Cousins et al., 2013) (henceforth, the vestibular identification threshold). Eye movements were recorded throughout the trials and, thus, the onset of the vestibular nystagmus during chair rotation provided a measure of the VOR threshold. There were three imagery conditions: (1) ‘congruent’ in which the direction of the imagined rotation was the same as the physical chair rotation (2) ‘incongruent’ in which the direction of the imagined rotation was opposite to the physical rotation (3) ‘neutral’ in which the subjects were instructed not to imagine anything. We predicted that the congruency of visual imagery ought to influence higher level vestibular thresholds. We further hypothesized that if top-down influences from imagery contents impinge upon the earliest stages of vestibular functioning then we should also observe the VOR reflex modulated by the congruency between imagery contents and the physical chair rotation. Additionally, we questioned whether imagery–vestibular interactions are mediated by a single mechanism or by distinct and partially dissociable systems. For instance, imagery effects on the vestibular sense may be driven via joint and parallel modulation of VOR systems in the brain stem and cortical multisensory substrates (e.g. the posterior parietal cortex). If this was the case, then, we ought to find that imagery influences upon the VOR and self-motion-related perceptual choices to be tightly correlated.

## Experimental procedure

### Participants

In the imagery experiment (i.e. Experiment 1) 16 naïve right-handed individuals participated in the study (nine female, mean age = 20.9 years, age range = 19–23 years). For the control experiment, directional cueing (i.e. Experiment 2) 10 separate naïve right-handed individuals participated in the study (seven female, mean age = 29.2 years, age range = 21–34 years). All of the participants had no previous history of vestibular, ophthalmological, neurological or psychological disorder. All participants provided informed written consent approved by the Charing Cross Hospital Research Ethics Committee.

### Vestibular stimulation

**Fig. 1:**
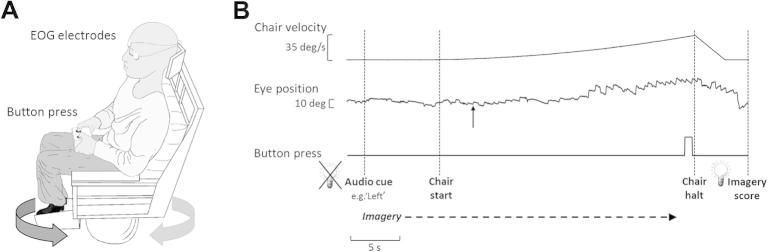
Experimental set-up and protocol. (A) The subject is seated in the dark on a vibration-free motorized rotating chair that can move rightward (light gray arrow) and leftward (dark gray arrow). Eye movements are recorded using EOG at all times and the perception of rotations is indicated by a two button press hand held device. (B) A recording of a single trial, which begins with the lights going off followed by an audio cue to inform the subject of the direction of the imagined rotation. The chair then starts rotating after 6–8 s with an exponential increase in velocity (note the chair velocity trace represents the true velocity of the chair). The subject indicates the direction of the chair rotation with a button press, following which the chair comes to a gentle halt. At the end of the trial, the lights are switched on and the subject is asked to rate the vividness of the imagery on a scale of 0–3. The onset of nystagmus is indicated by a top pointing arrow.

For both experiments 1 and 2 the vestibular stimulus was identical. Subjects were seated on a vibration-free motorized rotating chair (Contraves, USA). The head was supported by a chin and occipital rest in the normal upright position to minimize any head movements. The experiment was conducted in total darkness with white-noise masking delivered via a pair of chair-mounted speakers positioned behind the subject’s head. Subjects held a controller with two push buttons (Fig. 1A). Rotations were performed in the horizontal (yaw) plane, with chair rotations starting from rest with an initial acceleration of 0.3°/s^2^, increasing by 0.3°/s^2^ every 3 s (Cutfield et al., 2011; Cousins et al. 2013). The incremental acceleration continued until a button was pressed, after which the chair decelerated to rest. In each trial, the chair reached a velocity of at least 9°/s (15 s of rotation), even if a button was pressed prior to this velocity being reached, in order to obtain consistent vestibular nystagmus.

### Experimental protocol

#### Experiment 1: Imagery experiment

Each trial started with an auditory cue (“left” or “right”) played through the speakers, which instructed the subjects to imagine themselves rotating to the left or right respectively (Fig. 1B). If no audio cue was played, the subjects were instructed not to imagine anything. Following the cue, there was a delay of 6–8 s (randomized) before the chair started to rotate to allow sufficient time for the imagery process to develop. Subjects were instructed to press a button with the right or left thumb as soon as they felt a sensation of physical rotation to the right or left respectively (Fig. 1B). Following the chair stop, lights were switched on to indicate the end of the trial and subjects were asked to rate the strength of their imagined rotation on a discrete scale of 0–3 (0 = no imagination, 1 = weak rotation imagery, 2 = strong rotation imagery, 3 = very strong rotation imagery resembling actual physical rotation). Each subject was given practice trials on the chair at the beginning of the experiment to familiarize them with vestibular stimulation and aid the process of imagery. Specifically this familiarization process entailed the subjects experiencing three physical rotations for both rightward and leftward directions using exactly the same velocity profile as per the main experiment. These practice trials allowed for the subjects to familiarize themselves with both the experimental context and allowed for them to actually experience the motion profile during physical rotation. Apart from this, there were no other specific instructions to the subjects regarding imagery.

Trials consisted of chair rotations in two directions (leftward and rightward) under three imagery conditions (‘left’ or ‘right’ rotation and no imagery). These conditions can be grouped on a single congruency factor, according to which the direction of the physical rotation of the chair and the imagined rotation are ‘congruent’ (if both referred to the same direction, namely, ‘left imagery’ followed by leftward chair rotation), ‘incongruent’ (if rotation imagery referred to a different rotation direction to the physical chair rotation) or ‘neutral’ (in the no imagery condition). Each participant performed 100 trials in total, which were randomized across trials. There were 20 neutral trials, 40 congruent and 40 incongruent, with an equal number of left and right physical chair rotations.

#### Experiment 2: controlling for directional cueing/response bias effects

To control for the possibility that any effects in experiment 1 were attributable to an attentional or response compatibility effects stemming from the presentation of the auditory presented imagery cues in experiment 1, we performed the following control experiment. This experiment was identical to experiment 1 except that participants were not required to imagine self-motion following the presentation of the auditory cues; instead participants were merely asked to remember the auditory cue throughout the trial for a memory test after completion of the perceptual vestibular response. So, each trial of this control experiment started with an auditory cue (“left” or “right”) played through the speakers (a no-cue condition was also included as in experiment 1) and participants were instructed to remember and recall at the end of the trial. Following the cue, there was a delay of 6–8 s (randomized) before the chair started to rotate to control for the time delay in the imagery experiment. Subjects were instructed to press a button with the right or left thumb as soon as they felt a sensation of physical rotation either to the right or left respectively. Following the chair stop, lights were switched on to indicate the end of the trial and subjects were asked to recall the cue.

Trials consisted of chair rotations in two directions (leftward and rightward) under three different cues (‘left’ or ‘right’ rotation and no cue). As in experiment 1, these conditions can be grouped on a single congruency factor, according to whether the direction of the physical rotation of the chair and the cue were ‘congruent’, ‘incongruent’ or ‘neutral’ (no-cue condition). Each participant performed 100 trials in total, which were randomized across trials. There were 20 neutral trials, 40 congruent and 40 incongruent, with an equal number of left and right physical chair rotations.

### Vestibular oculo-motor threshold measurement

Horizontal eye movements were recorded throughout experiment 1 only using DC-coupled bitemporal electrooculography (EOG) (Fig. 1). Prior to the start of the experiment, calibration of eye position was obtained by instructing the subject to fixate targets appearing at 20° displacements to the right and left of the visual field. EOG, push buttons and chair tachometer velocity signals were sampled at 250 Hz. The oculomotor thresholds were found by measuring the chair velocity required to generate consistent vestibular nystagmus, as previously described (Cutfield et al., 2011; Cousins et al. 2013). Eye position data were first differentiated and then de-saccaded, that is, saccades were identified using previously published eye acceleration criteria (Seemungal et al., 2004) and then filtered out. The slow phase velocity (SPV) was derived using custom-made analyses program (Analysis; Mr. D Buckwell) using both eye displacement and eye velocity data. The onset of nystagmus was determined to occur at the offset of the first nystagmic saccadic beat (fast phase) and when the SPV consistently departed from baseline values.

### Vestibular perceptual threshold measurement

For both experiment 1 and 2, vestibular perceptual threshold was determined by measuring the time taken from the onset of chair acceleration to the button press and represents the velocity in °/s at which the participant could identify the rotational movement.

Data for both experiments were analyzed by means of Repeated-measures ANOVA in SPSS.

## Results

### Experiment 1: imagery

We conducted a 2 (Rotation: right or left) × 3 (Imagery Condition: neutral, congruent, incongruent) repeated measures ANOVAs on both the vestibular–ocular reflex and perceptual threshold data.

#### Vestibular–ocular reflex (VOR) thresholds

**Fig. 2:**
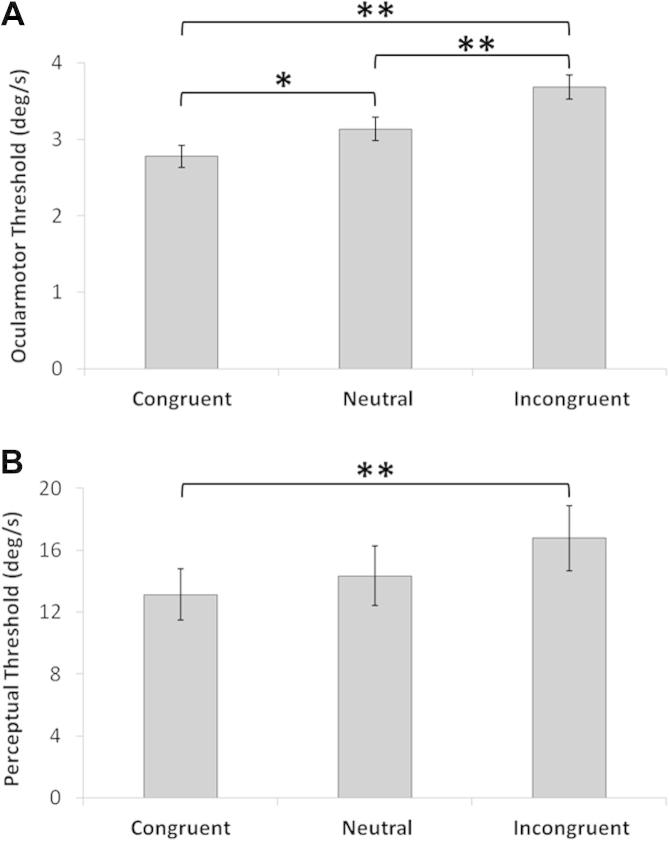
Effect of different imagery conditions on vestibular–ocular reflex and perceptual thresholds. (A) The oculomotor threshold was significantly reduced if the imagined rotation was in the same direction as the chair rotation (i.e. congruent) compared to the condition in which no imagery was present (i.e. neutral). In contrast, if the imagined rotation was in the opposite direction to the chair rotation (incongruent) then the oculomotor threshold was increased. (B) A statistically significant increase in perceptual threshold was found between the incongruent and incongruent conditions, which was also present for the oculomotor thresholds (A).

There was no effect of rotation direction [*F*(1,15) = 2.21, *p* = 0.16] but a significant effect of condition [*F*(2,30) = 19.44, *p* < 0.001]. Pairwise comparisons with Bonferroni correction showed a significant reduction of the VOR threshold in the congruent condition (mean = 2.78°/s, SD = 0.84°/s) relative to neutral (mean = 3.14°/s, SD = 0.87°/s; *p* = 0.031) and the incongruent conditions (mean = 3.69°/s, SD = 0.88°/s; *p* < 0.001) (Fig. 2A). In contrast, the oculomotor VOR threshold in the incongruent condition was significantly increased compared to the neutral condition (3.69°/s versus 3.14°/s, *p* = 0.003; Fig. 2A).

#### Vestibular perceptual thresholds

The number of incorrect responses, that is, button presses not matching the physical movement of the chair, across all subjects and trials was noted. Only one mistake was made in the neutral condition, three in the congruent condition and eight in the incongruent condition. All incorrect responses were discarded from the analysis.

There was no significant effect of rotation direction [*F*(1,15) = 0.26, *p* = 0.62]. The effect of condition was significant [*F*(2,30) = 5.41, *p* = 0.01]. Pairwise comparisons with Bonferroni correction revealed that perceptual threshold was significantly higher in the incongruent condition (mean = 16.78°/s, SD = 11.91°/s) compared to the congruent condition (mean = 13.14°/s, SD = 9.42°/s; *p* < 0.001; Fig. 2B).

**Fig. 3:**
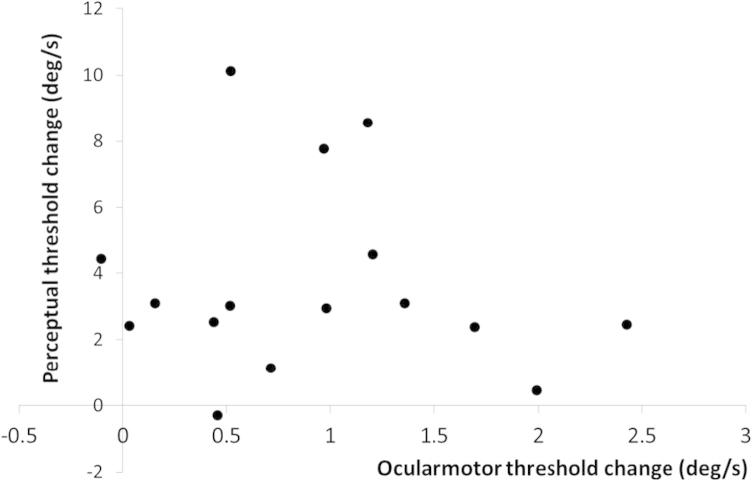
Plot illustrating the absence of correlation between the imagery congruency effect on perceptual measures (i.e. incongruent – congruent scores) and the imagery congruency effect on oculomotor measures.

The imagery congruency was found by calculating the difference between the perceptual or oculomotor measures in the incongruent and congruent conditions. Notably, imagery congruency effects on both VOR and perceptual thresholds were clearly uncorrelated (*r* = 0.012; Fig. 3).

Hence, for both oculomotor and perceptual responses, thresholds were found to be elevated in the incongruent condition compared to the congruent condition.

#### Mental imagery score

We conducted repeated-measures ANOVAs on the mean rotation imagery scores (=how strongly subjects rated their imagery vividness) with rotation direction (left, right) and imagery conditions (congruent, incongruent) as factors. There was no significant effect of rotation direction [*F*(1,15) = 0.76, *p* = 0.40] but a significant effect of imagery condition [*F*(1,15) = 4.89, *p* = 0.043], with higher rotation imagery scores in congruent (mean = 1.93, SD = 0.42) relative to the incongruent condition (mean = 1.79, SD = 0.42)(*t*(15) = 2.51, *p* = 0.018).

### Experiment 2: controlling for directional cueing/response bias effects

Recall here that this experiment was performed to discard the possibility that the congruency effects reported in experiment 1 could be due to attention or response bias effects triggered by the presentation of the auditory cues (i.e. that an exposure to a ‘left’ auditory cue might have facilitated responses for ‘left’ rotations relative to ‘right’ rotations, namely, a congruency effect). This experiment was identical to experiment 1 except that participants were not required to imagine self-motion following the presentation of the auditory cues. They were merely asked to remember the auditory cue throughout the trial for a memory test after completion of the perceptual vestibular response.

The number of incorrect responses, that is, button presses not matching the physical movement of the chair, across all subjects and trials was noted. No mistakes were made in the neutral condition, 1 in the congruent condition and 4 in the incongruent condition. All incorrect responses were discarded from the analysis. Furthermore, any trial in which the subject either forgot the cue or remembered the cue incorrectly was discarded from the analysis. There were only four such trials across all subjects.

**Fig. 4:**
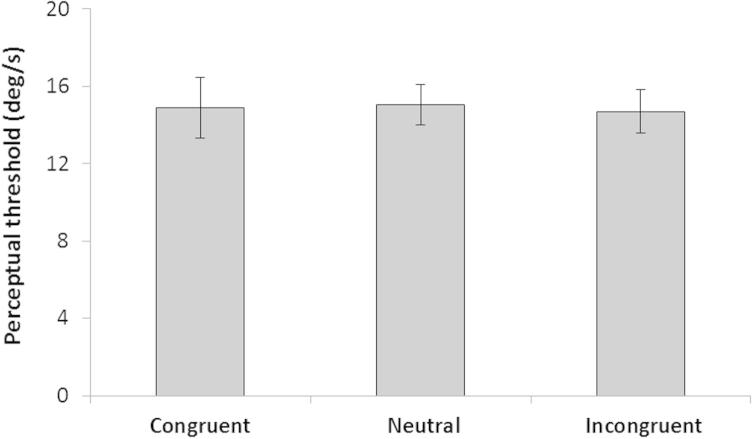
Effects of attentional cuing upon vestibular perceptual thresholds. As can be observed, there was no difference in perceptual thresholds for either the congruent or incongruent conditions when compared to each other or the condition with no attentional cue (i.e. neutral condition).

A 2 (Rotation: right or left) × 3 (cuing: no cue, congruent, incongruent) repeated measures ANOVAs showed no main or interaction effects on for the vestibular perceptual thresholds (*F*(1,9) = 0.45, *p* = 0.55, for the effect of rotation direction and *F*(1,9) = 0.57, *p* = 0.81, for the cuing effect; Fig. 4). The mean perceptual threshold values for the trials were 15.06°/s during no cue, for congruent cues 14.89°/s and for incongruent cues 14.70°/s. These results indicate that exposure to the auditory cues alone did not trigger any significant attention or response bias effect in the perceptual vestibular response. Thereby, these patterns of results suggest that the effects reported in experiment 1 were indeed related to imagery.

## Discussion

We provide a novel demonstration of the interaction between imagery and vestibular processing. The results demonstrate that mental imagery can shape angular self-motion perception. The ability of the observers to identify themselves rotating in the chair rotation was influenced by the contents of imagery. When the direction of the imagined rotation was incongruent with the physical rotation, a higher velocity of the chair was required for participants to experience self-motion relative to imagery congruent trials, in which the imagined rotation matched the physical chair rotation.

Interestingly, we found that ratings of the vividness of the imagined rotations were likewise influenced by the match/mismatch to the direction of physical rotation. For instance, the vividness of imagery was higher when the observers were physically rotated in the same direction as the imagined content relative to when the physical and imagined rotations were incongruent. Previous studies indicate that both visual imagery and a mental rotation are affected by vestibular stimulation (Mast et al., 2006). These results illustrate the bidirectional relationship between imagery and self-motion.

Previously, it has been shown that during passive rotatory head-body accelerations as employed in the present study, covert attention was shifted in the direction of rotation and the direction of the fact-phase of the vestibular nystagmus (Figliozzi et al., 2005). It could be argued that such modulation of spatial attention, driven by the direction of physical rotation, was also present in our study. Note, however, that the modulation of the VOR in the present study was not driven by the direction of rotation; there were an equal number of trials for left and rightward physical rotations in both the congruent and incongruent imagery conditions, so the imagery effect on the VOR was driven by the imagery-physical rotation congruency independently from the direction of rotation. Therefore, our demonstration of how imagery can exert effects upon the VOR and vestibulo-perceptual thresholds is distinct from the effects of physical rotation on spatial selection processes as previously reported. Moreover, by keeping the buttons and the response mappings the same in both Experiment 1 (imagery experiment) and Experiment 2 (direction cueing), the effects observed upon vestibular processing were only observed in Experiment 1, directly ruling out any spatially driven response compatibility effects.

Similar to here (Mertz et al., 2000) showed that same direction self-motion imagery improved the recognition of the actual linear acceleration while the opposite direction conditions degraded the recognition rates. However our study employed *angular* vestibular stimulation that activates the vestibular system only (via semicircular canals) while passive linear accelerations employed in Mertz et al. involve a number of sensory streams including vestibular otoliths, the somatosensory system and truncal graviceptors (Mittelstaedt, 1996). The exquisite vestibular selectivity of rotational thresholds (Seemungal et al., 2004; Cutfield et al., 2011) is in stark contrast to the multisensory nature of the process involved in detecting linear accelerations, as underlined by the fact that in some studies employing linear acceleration (Gianna et al., 1997) and tilt thresholds (Bisdorff et al., 1996) are not completely abnormal in patients with bilateral loss of vestibular function. Further support for this viewpoint is provided by a recent comprehensive dataset that demonstrates in patients with bilateral vestibular failure, thresholds for yaw rotations are considerably more abnormal than those found during *y*-translations (Valko et al., 2012). Therefore, our study is probably the first to show that mental imagery can modulate vestibularly mediated self-motion perception.

Most notable is the finding that imagery contents impinged upon the earliest stages of vestibular processing, namely, influencing the VOR thresholds in both facilitatory and inhibitory ways. VOR thresholds increased when the direction of the imagined rotation was incongruent with the physical rotation, relative to the neutral baseline. Conversely, when the imagined self-rotation was congruent with the physical rotations, the threshold for VOR was reduced relative to the neutral baseline. This finding indicates that the effects of imagery on self-motion perception reflect the operation of top-down processes that permeate the low-level vestibular reaction to physical rotation. Hence, the vestibular sense, alike other sensory processes (e.g. vision), is susceptible to modulation by higher order cognitive processes associated with attention, working memory or mental imagery (Gazzaley and Nobre, 2012; Soto et al., 2005; Soto et al., 2008). In line with this, it has recently been shown that higher order processes such as bistable perception during binocular rivalry or visual-spatial attention processes can modulate low level brain structures (Arshad et al., 2013a) including the VOR (Arshad et al., 2013b).

A number of brain-imaging studies suggest common neural networks for mental imagery and perception in various sensory modalities (Kosslyn et al., 2001). In the vestibular sense, two main approaches have been implemented. Firstly the recall of the sensation of rotation on a chair has been associated with widespread cortical activation predominantly in premotor areas (zu Eulenburg et al., 2013) involved in action control and action-orientated mental imagery (Palmiero et al., 2009) but not vestibular cortical areas (zu Eulenburg et al., 2013). Secondly, tasks requiring mental rotation of human bodies in space are also known to activate motor areas as found by Zu Eulenberg et al., but additionally it activates neural correlates typically associated with vestibular processing namely, posterior insula (PIVC), intraparietal sulcus, parietal operculum and the inferior parietal lobules (Dieterich et al., 2003; Lopez and Blanke, 2011; zu Eulenburg et al., 2013; Hitier et al., 2014). Moreover, it has recently been demonstrated that following vestibular dysfunction, namely due to acute vestibular neuritis or BPPV, it leads to an impaired ability to perform mental rotation, either of one-self or human figures (Candidi et al., 2013), implying that abnormal peripheral vestibular inputs can directly influence the underlying cortical processes associated with imagery via bottom-up processes (Candidi et al., 2013). Hence, it is possible that the interaction between imagery and vestibular perception is supported by overlapping, multisensory cortical areas (e.g. parietal) (Candidi et al., 2013), and that the dual activation observed for vestibular and pre-motor areas during imagery enables for the updating of spatial reference frames (Zacks, 2008).

Despite oculomotor and perceptual thresholds both being modulated in similar directions by the different imagery conditions, the degree of the modulations did not correlate. One interpretation of this result is that, although imagery–vestibular interplay may be mediated by a cortical multisensory substrate, there may be additional distinct and to some extent independent mechanisms through which mental imagery shapes self-motion perception. We note that whether vestibular perception shares the same neural mechanism as oculomotor processing underlying the VOR remains disputed (Shaikh et al., 2013). The processing of the two has been uncoupled under certain circumstances in both healthy individuals and in patients (Clement et al., 2008; Seemungal et al., 2011; Cousins et al., 2013). For example, during adaptation to repeated vestibular stimulation, the extent of the habituation differed between the VOR and perceptual responses (Guedry et al., 1992; Merfeld et al., 2005; Clement et al., 2008; Nigmatullina et al., 2013). Hence it is possible that imagery congruency effects on the vestibular sense operate through distinct mechanisms, namely, an ‘early’ mechanism associated with brain-stem VOR-related pathways and a ‘late’ mechanism operating on cortical representations that are used for computing multisensory perceptual choices. Future studies ought to fully characterize the neural bases that mediate the interaction between mental imagery and self-motion perception. Finally, our results can have practical clinical implications in the rehabilitation of patients with vestibular symptoms. Although it has been hypothesized that self-motion mental imagery might be helpful in the process of vestibular rehabilitation for dizzy patients (Lopez et al., 2011) current rehabilitation protocols, including those incorporating cognitive behavioral therapy (Andersson et al., 2006; Edelman et al., 2012), do not make use of self-motion imagery to counteract subjective symptoms of rotation. Our data in normal subjects suggest that the effects of imagery in patients undergoing vestibular rehabilitation should be investigated.
